# Supplementary material for: Causal speech enhancement using dynamical-weighted loss and attention encoder-decoder recurrent neural network
Source: PLoS One. 2023 May 11;18(5):e0285629. doi: 10.1371/journal.pone.0285629 (PMC10174555; doi:10.1371/journal.pone.0285629)
Supplement: Attachment — Submitted filename: Response to Reviewers.pdf [file pone.0285629.s004.pdf]

The Editor in Chief
PLOS ONE Journal

Subject: **Response to Reviews**

The authors are thankful that the Editor in Chief, PLOS One Journal and the respected reviewer for their valuable inputs to improve the technical contents of the study. The authors have seriously taken actions to answer and address the valuable concerns. The changes of the **Reviewer#1** are highlighted with RED color where for **Reviewer#2** the comments re highlighted with BLUE color.

Reviewer #1: Causal Speech Enhancement using Dynamical-Weighted Loss and Attention Encoder Decoder Recurrent Neural Network" is a good topic for paper but having some suggestions

1- Compare results with some current developed methods and use those references in your introduction.

Response: Thank you for the important suggestion, the results are compared to the recently developed methods and the reference methods are reflected in the introduction part of the revised manuscript. The Tables are revised to address the suggestion of respected reviewer. Table captions are highlighted with Red to show the changes.

2- Use one more different input data for analysis and evaluation.

Response: Thank you for the important suggestion. This algorithm was intended for the additive noisy backgrounds; however, to address the concern of the respected reviewer, the authors have included noisy reverberation as other input data.

Action: The following text and results are added to the revised paper.

This section examined the dereverberation performance of the proposed SE. To train the SE model, three reverberation times (0.4 sec, 0.6 sec, and 0.8 sec) are considered. A total of 100 anechoic speech utterances from the IEEE dataset [40] are used to create the training dataset. The testing dataset contains 40 reverberant speech utterances. There is no overlapping between the speech utterances used during model training, and testing. The proposed method with reverberant speech utterances are compared and examined for the dereverberation. The results are compared with study of Wu and Wang [49], where estimated inverse filters and spectral subtraction are used to reduce the reverberation. Table 11 shows the results using STOI and PESQ. The proposed method delivered the best STOI and PESQ scores, i.e., STOI≥78.3%, and PESQ≥2.45 at RT≥4 sec. The spectrograms are provided in Fig. 8 where the smearing energy produced by reverberation is considerably reduced, showing that the reverberation performance of the proposed method.

Table 11. Performance analysis of proposed SE in reverberant situations.

Metric	Method	Reverberation Time		
		0.4 sec	0.6 sec	0.8 sec
STOI	Noisy Reverb	53.1	48.5	40.2
	Wu and Wang [49]	65.3	59.8	55.1
	Proposed	78.3	70.1	68.4
PESQ	Noisy Reverb	2.11	1.98	1.81
	Wu and Wang [49]	2.32	2.00	1.92
	Proposed	2.45	2.19	2.04

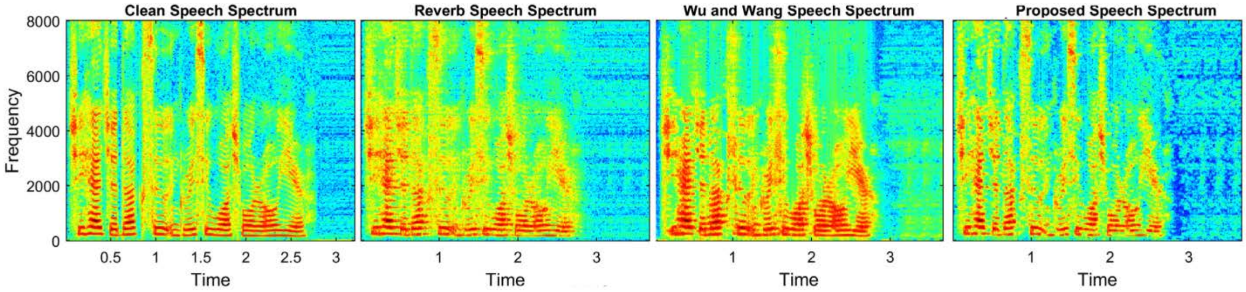

Figure 8: The time-varying spectral analysis of speech utterances processed by Wu and Wang [49] and the proposed speech enhancement algorithm.

3- Use proper references.

Response: Thank you for the suggestion, the references is arranged in the proper manner to address the concern.

Reviewer#2: The manuscript is interesting; however, the following comment should be addressed

1 – Please include problem statement.

Response: Thank you for the important suggestion, the problem statement is included in the revised manuscript.

Action: Speech enhancement (SE) reduces background noise signals in target speech and is applied at the front end in various real-world applications, including robust ASRs and real-time processing in mobile phone communications. SE systems are commonly integrated into mobile phones to increase quality and intelligibility. As a result, a low-latency system is required to operate in real-world applications. On the other hand, these systems need efficient optimization. This research focuses on the single-microphone SE operating in real-time systems with better optimization.

2 - Improvement ratio between the proposed and existing works should be included.

Response: Thank you for the suggestion, the improvement ratios between the proposed and related studies are included in the revised manuscript.

3 – In the Introduction, the authors need to refer to other speech enhancement algorithms such as : i) doi: 10.1088/1757-899X/1090/1/012102, ii) doi: 10.3390/s21217025, and ii) doi: 10.1109/ACCESS.2019.2929864.

Response: The introduction part is modified with the references suggested. Thank you

4 – The contribution should be included as a list for better readability.

Response: The contributions are listed in the revised manuscript to address the concern of the reviewer. Thank you

5 – Please check the numbering of the subsections.

Response: Thank you for the correction, the sections and subsections are corrected in the revised manuscript.

6 – Define the functions used such as “|·|” , “||·||”, “*” , etc .

Response: Thank you for the important correction, the typos in equations is corrected and these notations are defined in the revised manuscript.

7 – The authors utilize STFT; however, there are different types of transforms which are based on orthogonal polynomials. The authors need to refer to the difference between the Fourier Transform and the following transforms Krawtchouk transform (doi: 10.3390/e23091162), Hahn Transform (doi: 10.1109/ACCESS.2022.3170893), and Meixner transform (doi: 10.1007/s11554-021-01093-z). This will help the researchers to utilize other transforms for SE.

Response: Thank you for the important suggestion, the authors have used STFT transform widely used in the speech signal processing. The other mentioned transforms are associated to the different applications and the authors will conduct a separate study based on these transforms. The mentioned transforms are added with the references to the revised manuscript.
